# Bending Properties of Zigzag-Shaped 3D Woven Spacer Composites: Experiment and FEM Simulation

**DOI:** 10.3390/ma12071075

**Published:** 2019-04-01

**Authors:** Liming Zhu, Lihua Lyu, Xuefei Zhang, Ying Wang, Jing Guo, Xiaoqing Xiong

**Affiliations:** School of textile and material engineering, Dalian Polytechnic University, Dalian 116034, China; zlm0501abc@163.com (L.Z.); zhangxuefei1026@126.com (X.Z.); wy104020@163.com (Y.W.); 13704091879@163.com (J.G.)

**Keywords:** zigzag-shaped 3D woven spacer composite, bending deformation and damage, finite element method, failure mechanism

## Abstract

Conventionally laminated spacer composites are extensively applied in many fields owing to their light weight. However, their impact resistance, interlaminar strength, and integrity are poor. In order to overcome these flaws, the zigzag-shaped 3D woven spacer composites were rationally designed. The zigzag-shaped 3D woven spacer fabrics with the basalt fiber filaments tows 400 tex (metric count of yarn) used as warp and weft yarns were fabricated on a common loom with low-cost processing. The zigzag-shaped 3D woven spacer composites were obtained using the VARTM (vacuum-assisted resin transfer molding) process. The three-point bending deformation and effects of damage in zigzag-shaped 3D woven spacer composites were studied both in experiment and using the finite element method (FEM). The bending properties of zigzag-shaped 3D woven spacer composites with different direction, different numbers of weaving cycle, and different heights were tested in experiments. In FEM simulation, the geometrical model was established to analyze the deformation and damage based on the 3D woven composite structure. Compared with data obtained from the experiments and FEM simulation, the results show good agreement and also prove the validity of the model. Based on the FEM results, the deformation, damage, and propagation of stress obtained from the model are very helpful in analyzing the failure mechanism of zigzag-shaped 3D woven composites. Furthermore, the results can significantly guide the fabrication process of real composite materials.

## 1. Introduction

The impact resistance, interlaminar strength, and integrity of conventionally laminated composites are poor. In order to overcome these flaws, 3D textile composites have been invented. The higher fracture toughness and interlayer shear strength of 3D textile composites contribute to the extensive investigations of composites in mechanical areas. Chen [1] presented an overview of the fabrication processes for many kinds of three-dimensional woven textile preforms for composites, pointing out that spacer fabrics are mainly made using knitting technology. Li [2] investigated the effect of pile height, pile distribution density, and pile structure on the spacer composite. The pile structures were both 8-shaped and corrugated. In addition, the E-glass fiber was employed for weaving. 3D woven spacer composites are a competitive alternative over other composites of 3D textile structures because of their numerous advantages, including their light weight, available structure, highest stiffness, strength, low fabrication cost, and high manufacturing efficiency. The applications of 3D woven spacer composites have been extended to aerospace, vehicles, as well as civil engineering, and other areas. Mountasir [3] utilized E-glass/polypropylene hybrid yarns to manufacture spacer composites that were intended for lightweight engineering applications. The results revealed that the processing of hybrid fibers and the geometry of woven structures had a strong effect on the mechanical properties of composites. Badawi [4] researched the weaving process of 3D spacer textiles and presented the technological aspects of the process, as well as relative work theory of common and special looms in detail. Gu et al. [5] studied the weaving principles and conditions of 3D spacer woven fabrics, and recommended the 3D integrity structure of the junction type of round, rectangle, and honeycomb in detail. Zigzag-shaped and X-shaped 3D spacer woven fabrics are regarded as examples to investigate the weaving principles and conditions for common looms. The weaving process of 3D spacer fabric was researched, whereas the properties of 3D woven spacer composites were not further researched. Liu [6] explored the effect of hypo-atmospheric pressure plasma on a three-dimensional woven fabric of glass fiber with an 8-shaped structure. The pretreatment time was found to have a significant influence on the material. The surface of the fiber—which improved interfacial adhesion between fibers and resin after the pretreatment—became coarse. Karahan [7] researched the effect of different core thicknesses and foam fillings on the low velocity impact characteristics of an 8-shaped sandwich composite. The results showed that impact damage was limited to the vicinity of the point of impact, and the integrity of the structure was not affected. The yarns used in the experiments were also E-glass fibers. Li [8] investigated the effect of the temperature and core heights on the bending properties of woven spacer composites. The bending properties at liquid nitrogen temperatures were more significantly improved than those at room temperature. This was due to the fact the stiffness and strength of the matrix and the fiber/matrix interface at liquid nitrogen temperature were improved significantly than those at room temperature. The core height had a great influence on the composites both at room temperature and liquid nitrogen temperatures. Matthieu [9] manufactured angle interlock (AI) structures with through-thickness binding. The results showed that the transverse component was the one that led to transverse matrix cracking in the weft yarn under tensile loading.

The work described above focused on the effects of different factors and analyzed the damage mechanism of composites. The analysis of damage mechanisms was based only on the deformation process of composites and the final failure statements were based on observations made by the naked eye; many details in this failure process have been neglected. However, FEM (finite element method) is an effective way to address these problems. Based on this model, the failure process can be observed in detail. Furthermore, the mechanism can be readily analyzed. Lv [10] used a common loom to fabricate basalt fabrics and the tensile and bending properties were predicted by applying an FEM model. The results demonstrated the validity of the model. Lv [11] researched the effect of different height beams on the T-shaped composites manufactured by the VARTM (vacuum-assisted resin transfer molding) process. The corresponding model was established to analyze the failure mechanism. Lv [12] fabricated honeycomb fabrics of a sandwich structure with different cross-sectional shapes on an ordinary loom by rational design. Then, the bending properties in different cross-sections of shape composites manufactured by the VARTM process were studied. The FEM simulation was applied to analyze the failure modes and good agreements were obtained in the end. This research provided a secure foundation for this paper. Zhang et al. [13] used ABAQUS/Explicit to calculate the impact damage evolution of the 3D angle-interlock woven composite. Compared with the results obtained from FEM and experiments, this confirmed that FEM was effective in simulating the experimental process, especially the load–deflection curves and failure patterns. Zhou et al. [14] studied the transverse impact deformation and damage of 3D composite tubes with circularly braided structures using experimental and numerical approaches. The FEM results showed satisfactory agreement with the experimental data.

The purpose of this paper is to study zigzag spacer composites. The bending properties of zigzag spacer composites were investigated under the use of different directions, different numbers of weaving cycles, and different height fabrics in the weaving process by rational design. Then, composites manufactured by the VARTM process were tested. Considering the research described above, FEM simulation was applied to analyze the composite. A geometrical model was established to investigate the deformation and damage based on a zigzag 3D woven composite structure. The deformation, damage, and propagation of stress obtained from the model are very helpful in analyzing the failure mechanism. 

## 2. Experiment

### 2.1. Design and Weaving of Fabrics

**Figure 1:**
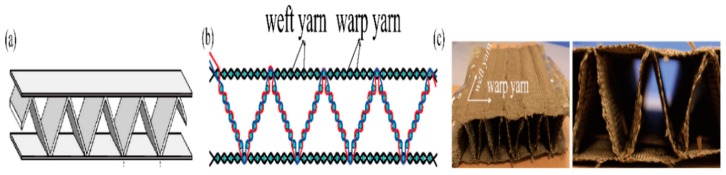
Different visual representations of zigzag-shaped spacer composites: (**a**) diagrammatic sketch; (**b**) cross-section; (**c**) real photograph.

The diagrammatic sketch of a zigzag-shaped 3D woven spacer composite is shown in Figure 1a. A cross-section of the zigzag-shaped 3D woven spacer fabric is shown in Figure 1b. The small circle indicates the weft yarns, and the lines indicate the warp yarns. The parameters of the zigzag-shaped 3D woven spacer fabrics with three different heights are shown in Table 1. The pictures of the zigzag-shaped 3D woven spacer fabrics are shown in Figure 1c. The filament tows of 400 tex basalt fiber (Zhejiang stone basalt fiber Limited by Share Ltd., Hengdian, China) were employed as warp and weft yarns.

The filament tows of basalt fiber purchased from the company were directly used in weaving without any pretreatment. The low twist of 10 twists per 10 mm has a good clustering effect on filament tows of basalt fiber. This is beneficial for the weaving of yarns and does not cause filaments to entangle. The spin capability of this fiber is good with the exception that the fiber is fragile in the vertical direction when weaving on a semi-automatic loom. Loom SGA 598 (from Jiangyin Tong Yuan spinning machine Co., Ltd., Jiangyin, China) was used for weaving. The zigzag-shaped 3D woven spacer fabric was composed of two surfaces and a connected layer which linked the top and bottom surfaces in addition to stabilizing the structure and were all constructed from plain woven fabric. The design and operation of plain weave method are simple and, in addition, the junctions between the three layers make the structures tight and stable. In order to weave the three layers, the top and bottom yarns, as well as intermediate connecting layer, need to be wrapped onto each of two warp beams. The lifting plan in this work was straight draw. In order to reduce the friction between filament tows of basalt fiber and reed, the reed counts were set to 30. Three layers were woven on the loom, and three shuttles were utilized to avoid floats. After weaving, zigzag-shaped 3D spacer fabrics were obtained with a width of 20 mm. The thickness of the layer was measured to be 0.4 mm using calipers. The parameters of the zigzag-shaped 3D fabrics with three different heights are shown in Table 1. The ability to manufacture 3D spacer fabrics on common semi-automatic weaving looms using rationally designed structures, rather than knitting looms, is considered advantageous. In this way, the composite structure becomes more stable.

### 2.2. Fabrication of Composites

Vinyl ester resin was used as matrix. Methyl ethyl ketone peroxide was used as solidification reagent, and cobalt was used as promoter. VARTM molding system was used for molding. The resin, relative solidification reagent and promoter were provided by Wuxi Qianguang Chemical Raw Material Co., Ltd. (Wuxi, China). The pump (GSG-2047), as well as positive and negative air pressure converter, were provided by Guangzhou Husong Electromechanical Trading Co., Ltd (Guangzhou, China) and Sino composite Co., Ltd (Shenyang, China), respectively. In fact, there were hollow positions in the fabric, suggesting that composite-manufactured, suitably sized molds should be selected. Also, after the resin is cured, the mold should be easy to remove. Thereby, the combination of cement and pasteboard was employed as the mold in this paper following many attempts. The cementitious prism was wrapped by the pasteboard and the size of combination was ensured, according to the hollow structure. Before the resin was impregnated, the fabric was supported by the combination mold. After the resin was cured, the combination mold was removed. Thus, the hollow structure was formed. Due to the molding method, VARTM, area where the resin was enriched were found in the walls of the intermediate zigzag-shaped layer. After adjusting the size of the mold, good progress was made toward addressing the problem. Finally, the height of composite was slightly higher than the height of supported fabric. In order to weave composites of different heights, different numbers of weft yarns were used to control the height when the zigzag-shaped intermediate layer of the fabric was fabricated, and the force used to control the beating-up was uniform. The height was measured using a ruler.

### 2.3. Testing of Composites

**Figure 2:**
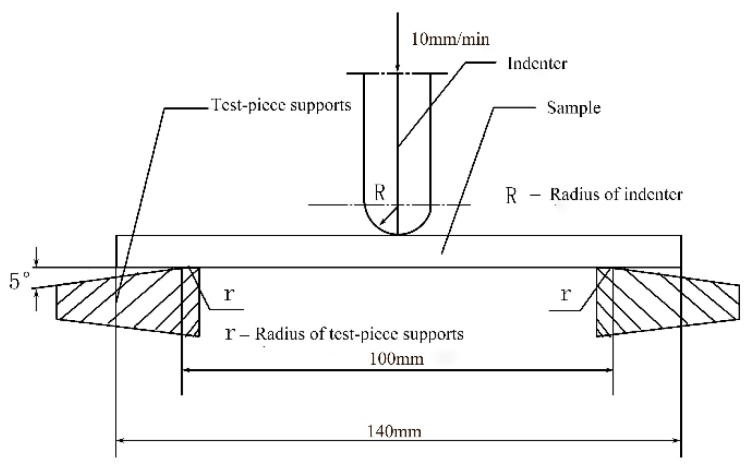
Schematic diagram of bending test.

Bending properties that were tested was according to GB/T1456-2005. Cutting samples were obtained from composite materials in zigzag and spacer wall directions. Then, a microcomputer control electronic universal testing machine (RGY-5 from Shenzhen Reger instrument Co., Ltd., Shenzhen, China) was applied for three-point bending test. The schematic diagram of the bending test is shown in Figure 2.

Figure 2 shows that the test speed is 10 mm/min, spacing is 100 mm, and sample length is 140 mm. In addition, the R is 5mm and the r is 2.5 mm. Also, the 3D woven spacer composites of zigzag shape with different thicknesses and numbers of weaving cycles were tested. The characteristics of the VARTM process are that the top surface of composite is slightly rough and the bottom surface is smooth. In order to decrease the experimental errors as quickly as possible, the samples employed in each test were cut from the same zigzag-shaped 3D spacer composite, and three samples of the same size were tested in each type experiment. Although the size of samples was same, the results differed slightly between the three samples. The best curve was selected and utilized in the paper. The effect of this factor on the bending property was not significant, and was therefore neglected in this paper.

## 3. Results and Discussion

### 3.1. Bending Load–Displacement Curves of Composites with Different Directions

**Figure 3:**
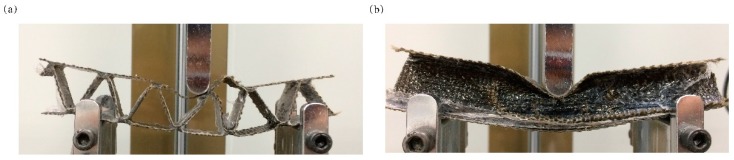
Tested results of (**a**) zigzag direction and (**b**) spacer wall direction.

The bending test of zigzag-shaped 3D woven spacer composites with zigzag and spacer wall directions are shown in Figure 3.

**Figure 4:**
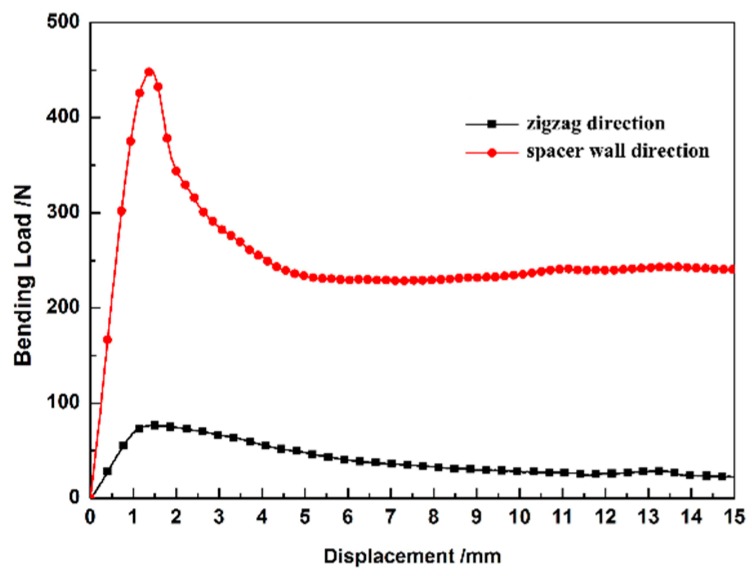
Bending load–displacement curves of zigzag-shaped 3D woven spacer composites with different directions.

In this part, the composite with height of 17 mm and single weaving cycle was utilized for testing. The direction of zigzag in the sample consisted of continuously zigzagging structures with a limited width of 25 mm and thin walls, as shown in Figure 3a. The width was set according to the width of the side length of the triangle in the spacer wall direction. That is, the width of the single weaving cycle. The sample was tested on the device, and it was observed that when the indenter of the universal testing machine touches the sample surface and gradually sinks, due to thin wall, the zigzag structure is then unable to support the surface against the load. In fact, the line where the indenter touches coincided with the line where the zigzag connects with the top surface. As the load grew, the direction of zigzags in the sample become bent overall. Due to the thinness of the top surface, the indenter easily slips, as shown in Figure 3a, followed quickly by destruction. The typical bending failure modes are compression failure in the upper surface, which is touched by the indenter; and tensile failure in the untouched bottom surface, as shown in the photograph. At the same time, the failure mode of the top or bottom surface can be confirmed by deformation of the composite after experiment and comparison with the original composite. The spacer wall direction sample is shown in Figure 3b. This sample was obtained after the composite was cut along the zigzag junction. The size of this sample is same as the zigzag direction sample except that it has a zigzag structure through the whole material as well as junctions which connect the bottom or upper surface and middle layer and stabilize the three layers. In bending tests, the surface of the spacer wall direction sample does not fail quickly. As the loads begin to affect the sample, the whole sample gradually bends until the zigzag structure begins to break. The bending loading–displacement curves of zigzag-shaped 3D woven spacer composites with different directions are shown in Figure 4.

The zigzag-shaped 3D woven spacer composites are elastoplastic. As Figure 4 shows, the curves have a peak load and clear tendency. It is obvious that the peak load value of the spacer wall direction is higher than that of the zigzag direction. The zigzag direction has a low peak load under the bending test. This is attributed to the thin face sheet structure. Compared with the spacer wall direction, the zigzag direction was not stable enough in the test. The slippage of indenter leads to the low peak load. The different maximum load values show that the structures of the zigzag and spacer wall direction samples have different damage performance under the same testing conditions. In Figure 4 it can be clearly seen that the zigzag-shaped 3D woven spacer composites are anisotropic. During the bending test process of spacer wall direction, it can be seen that the zigzag structure of 3D woven spacer composites shoulders the main bending load. Hence, the zigzag structure with spacer wall direction can obviously enhance the capability against the load by comparing the load–displacement curves of zigzag and spacer wall direction. In comparing the analysis of bending load–displacement curves with either zigzag and spacer wall directions in Figure 4, it is observed that the bending performance of the spacer wall direction material is closely relevant to the structure of the connected layer. Therefore, this composite was essentially designed to tolerate a major bending load in the spacer wall direction. The composite samples employed in the next experiments were all tested in the spacer wall direction.

### 3.2. Bending Load–Displacement Curves of Composites with Different Numbers of Weaving Cycle

**Figure 5:**
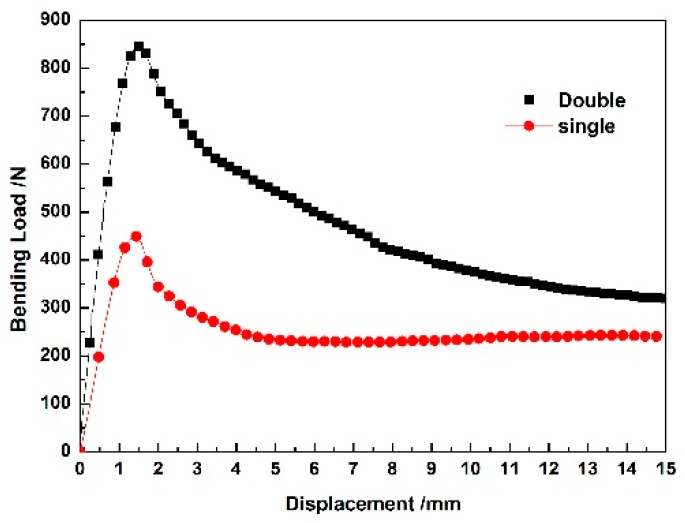
Bending load–displacement curves of zigzag-shaped 3D woven spacer composites with different numbers of weaving cycles.

There is a similar tendency for the curves of single and double weaving cycles in Figure 5, which indicates that the zigzag-shaped 3D woven spacer composites have the same failure mode. The cross-section of the zigzag-shaped 3D woven spacer composites consists of a row of triangles. Each structure with triangle cross-section is regarded as one weaving cycle. After cutting, the single and double weaving cycles were obtained. The single and double weaving cycles are the number of the structure, as Figure 3b shows. The cross-section of a single weaving cycle is a triangle structure tested in the spacer wall direction. The cross-section of double weaving cycles means that two triangle cross-section structures are tested under the same condition. With the number of zigzag structures increasing, the material can, in theory, tolerate greater loads. Thus, the peak load should be higher. The experiment attempts to explore whether the number of weaving cycles has any effect on the composite. In contrast to the bending load of a single weaving cycle, the bending property of double weaving cycles is obviously much better than the single one. This is because the zigzag-shaped 3D woven spacer composite with double weaving cycles has more supported parts and can distribute loads on more thin walls, meaning it can bear a bigger bending load than the single one. In other words, it also demonstrates that the bending load is interrelated to the number of weaving cycles. Therefore, increasing the number of weaving cycles is beneficial to improving the bending property of the composite.

### 3.3. Bending Load–Displacement Curves of Composites with Different Heights

**Figure 6:**
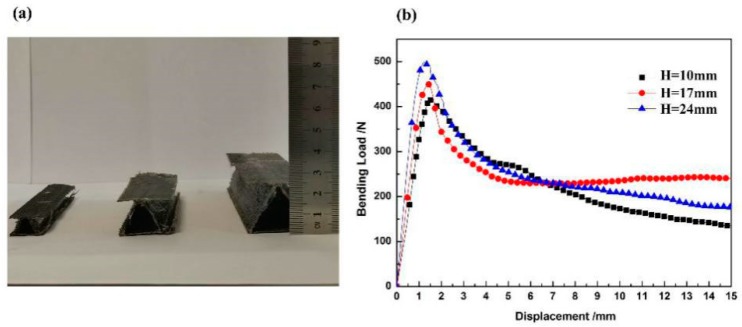
(**a**) The three different height composites. (**b**) Bending load–displacement curves of zigzag-shaped 3D woven spacer composites with different heights.

**Figure 7:**
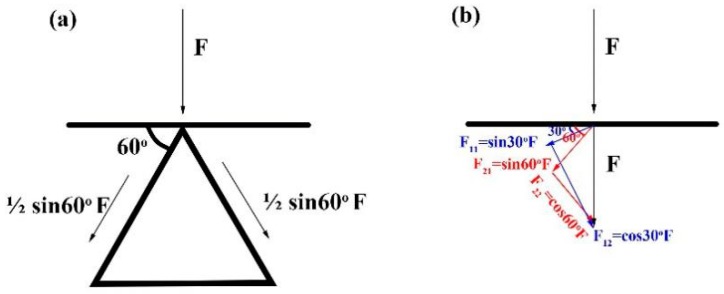
(**a**) Schematic diagram of load analysis of zigzag-shaped 3D woven spacer composites; (**b**) specific analysis diagram.

The bending load–displacement curves of zigzag-shaped 3D woven spacer composites with three different heights are shown in the Figure 6. The height is defined as the distance between the top–bottom surfaces. The three curves had similar tends, in other words, their failure modes were the same. The 24 mm high sample has the biggest bending peak load value among the three spacer heights, which corresponds with the best bending property. The bending peak load value of the sample with 17 mm height is medium, and the bending load value of the 1 mm high sample was the lowest. As a result, the greater the height of the zigzag structure, the bigger the bending load of the composite. Comparison of the three composites of different height, shows that the higher structures have a longer intermediate layer. This longer intermediate layer bears a greater load. The slope of the zigzag spacer walls was also seen to increase in the horizontal direction as the height increases. After measurement, the angles of the three different samples shown in the Figure 6a are 50°, 60° and 70°. Figure 7 is the schematic diagram of load analysis. Figure 7a presents the load analysis of zigzag structure in the bending test while Figure 7b is the specific analysis. As the angle increasing, the zigzag wall can undertake a greater load along the wall direction (F_21_ > F_11_) while the dividing force that is vertical with zigzag wall gradually decreases (F_22_ < F_12_). In fact, it is the dividing force that causes the composite failure. Hence, the structure becomes more stable and the bending property of the composite improves with the increase of height. The more stable the structures are, the higher the resulting peak load.

### 3.4. Bending Properties of Composites: Experiment and FEM Simulation

**Figure 8:**
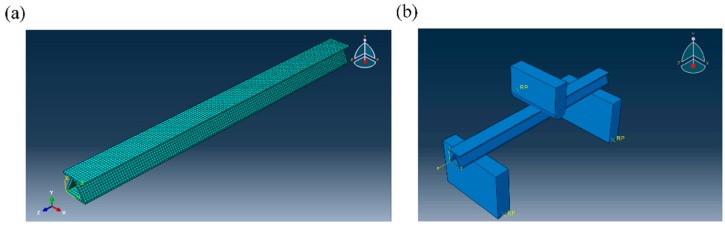
(**a**) Geometrical mesh model and (**b**) assembly model of the zigzag-shaped 3D woven spacer composite.

To exhibit the failure mode and mechanism of the zigzag-shaped 3D woven spacer composites precisely, ABAQUS software (6.14 version) was adopted to simulate the three-point bending test of 3D woven spacer materials. The geometrical models of the zigzag-shaped 3D woven spacer composites were established according to the actual specimen using an explicit module in a dynamic and explicit way. The composite was set as 3D deformable solid and the other touched parts were set as rigid. The composite model established in the job was an integrity. Thus, a local coordinate system and material orientation were assigned to the model. The displacement constraint of 15 mm was assigned to the indenter along the normal direction. The two bearings were set as fixed support. The property of the composite model was set as elasticity and plasticity. The geometrical beam model of sample with a thickness of 1 mm is shown in Figure 8a. For approaching the actual testing conditions, more touched parts were employed, as shown in Figure 8b.

The material properties of the zigzag-shaped 3D woven composites obtained from the simulation are shown in Table 2. The C3D8R solid element was utilized for the geometrical model. The size of the composite was set as 0.5 mm. The size of other rigid parts was set according to default. The total number of elements of the mesh model with a thickness of 1 mm is 5600; mesh model with 17 mm thickness is 11,200; and mesh model with 24 mm thickness is 15,680.

**Figure 9:**
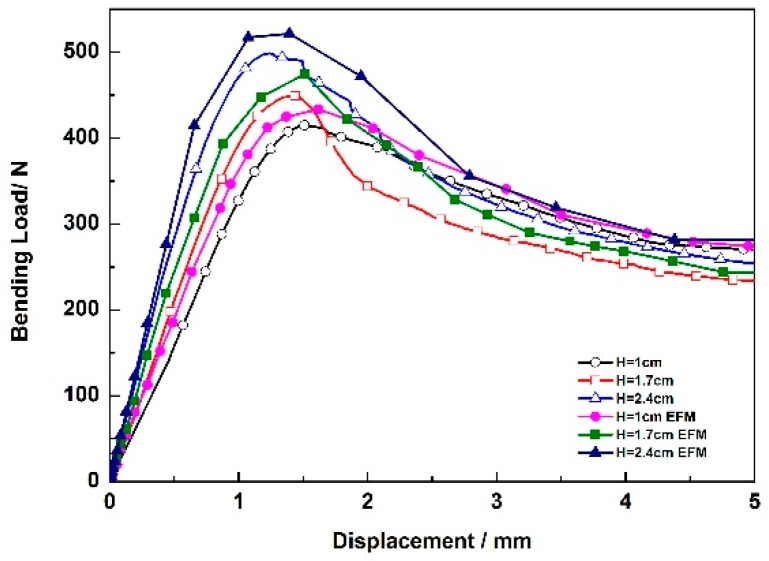
Bending load–displacement curves of the zigzag-shaped 3D woven spacer composites: Experiment and FEM simulation.

The bending load–displacement curves corresponding to the zigzag-shaped 3D woven spacer composite: experiment and FEM simulation of the different height composite are shown in Figure 9.

From Figure 9, it is obvious that the simulated curves have a similar tendency as the experimental results and the good agreement between these results demonstrates the validity of the FEM models. The displacement of yield point of all materials is similar. The increasing height makes the structure more stable. Thus, this means there is a different peak point for each of the materials. Of course, two kinds of curves have many differences, especially in certain locations, such as yield point and destruction point, and the FEM curves are slightly higher than the experimental curves. These differences are attributed to the self-defect of the zigzag-shaped 3D woven spacer composites. During the fabricating process of 3D woven spacer composites, errors and limits due to environmental conditions may cause many defects in the body of the resulting material. The actual conditions for the composite are complicated, such as weaving and formation of the composite. For example, some bubbles may be present in the resin when it is impregnated and have an adverse effect on the formation of composite. However, the simulated models represent ideal conditions, and many factors are omitted. Thus, the simulated results are better. In summary, the simulated curves can approach and forecast the experimental results, but the simulated results are slightly higher than the experimental results.

It can be seen in Figure 9 that the curves are obviously divided into three stages. In the first stage, the curve is approximately linear. Overall, the composite tolerates the load until it increases to the peak load. After reaching the peak load, the second stage starts. In this stage, the load is relatively stable and decreases slightly, due to the fracture of matrix that occurs in this stage. Subsequently, the load decreases quickly in the third stage, due to the breakage of yarns.

### 3.5. Failure Mode and Mechanism

**Figure 10:**
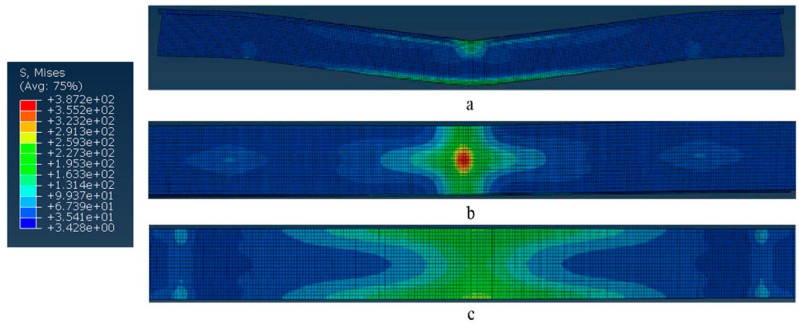
Equivalent stress photographs of bending FEM simulation at 0.6 s: (**a**) profile; (**b**) top surface; (**c**) bottom surface.

The equivalent stress photographs of bending FEM simulation at 0.6 s are shown in Figure 10. As the bending load gradually increases, the indenter gradually sinks. Following the model, it eventually becomes bent. The mise stress is an automatic output of the stress result by ABAQUS software. From Figure 10a, it is evident that the top and bottom surfaces of the model generated some changes. The touched area of top surface was compressed and the bottom surface is convex. This is a good proof demonstrating the failure of experiment. On the top surface, from Figure 10b, the major bending stress is centralized around the touched areas between the indenter of the universal testing machine and the sample. The bending stress unevenly extends along the top surface and is distributed in a cross. Furthermore, the max bending stress occurs in the top surface’s center, that is, the wall junction of the zigzag structure—this is the failure point. It indicates that the zigzag structure bears the main bending stress and the stress extends along the direction of junction. From Figure 10c, it is observed that the distribution of bending stress of the bottom surface differs from the top surface. Its characteristics are that the distribution of stresses narrows gradually from the edge to center, like an hourglass. Since the bending stress of the top surface spreads to the bottom surface along the walls, the stress spreads to other places such that the distribution of bending stress in the junction area of the bottom surface is wider than at the center.

**Figure 11:**
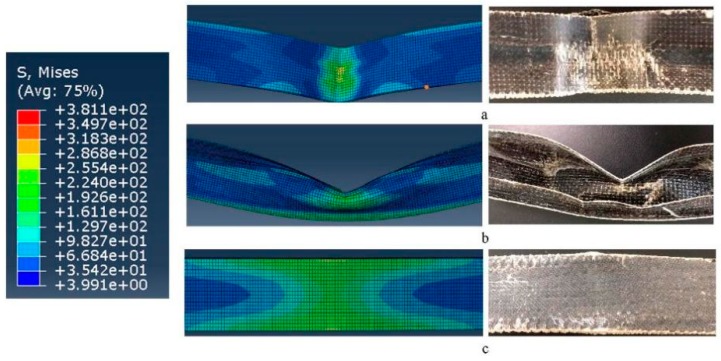
Equivalent stress photographs of bending FEM simulation and experimental samples of 3D woven spacer composite: (**a**) top surface; (**b**) profile; (**c**) bottom surface.

Figure 11 is the finally deformed comparison results of the bending FEM simulation equivalent stress photographs and experimental samples of 3D woven spacer composite. The equivalent stress photographs of bending FEM simulation and the experimental results of 3D woven spacer composite are similar and reveals that the simulation process can display the experimental process to some extent. Specifically, the top surface tolerates the compression and, consequently, exhibits downward indentation, as is shown in the Figure 11a. According to the comparison of pictures, the indentations differ, and there is overall indentation in the simulated model, but the indentation in the experimental sample is centered around the touched regions, between the indenter of the machine and the sample. The difference is caused by the model being set up to consider the material as homogeneous, when the experimental sample is actually heterogeneous. Then, the connected layers are compressed and generate an outward convex shape, as shown in Figure 11b. There are the same changes both in the simulated model and the experimental sample. The bottom surface shoulders the tension such that the bottom surface has slight bending, and the bending stress is concentrated upon the middle of sample, as shown in Figure 11c. Similar deformations are observed in the simulated model and the experimental sample.

To sum up, the simulated model and the experimental sample have a similar failure mode, which indicates that good agreement between the simulated model and experimental sample was obtained. Furthermore, the feasibility of using FEM models was also demonstrated.

## 4. Conclusions

The three-point bending performance of zigzag-shaped 3D woven spacer composites with different directions, different numbers of weaving cycle, and different heights were studied. The results indicate that the materials are fit to be tested in the spacer wall direction, because of their zigzag structure, which bears the main load. Moreover, with a higher number of weaving cycles, the structure of samples becomes more stable. Also, the bending properties of spacer composite are effectively enhanced. In addition, to some extent, the zigzag sample produced using a higher number of weaving cycles has better bending properties due to the increasing slope of zigzag layer walls. Following the above suggestions can greatly improve the bending property of designed structures. 

In addition, the thickness of the wall and the number of layers has a great effect on the properties of the composite. Also, improving the through-thickness connections between the zigzag layer and the top–bottom layers, and changing the orientations of fibers plays also contributes to the properties of the structure. The effects of these factors on the structure of the composite should be studied further in future work. Finally, the prediction of bending conditions and a failure model for experimental samples was achieved by adopting a FEM simulation model, and the similarity with experimental results demonstrates the feasibility of using the FEM simulation model.

The design of spacer composites aims at decreasing the weight of material and improving properties. Considering the research above, this work can be used to promote the usage of spacer composites on the roof of shanty houses, aircraft wings, and others, since the obtained composites present a much lighter alternative with better integrity than the materials used nowadays.

## Figures and Tables

**Table 1:** Parameters of the zigzag-shaped 3D fabrics with three different heights.

Cross-Sectional Shapes	Heights (mm)	Warp Density (yarn per 100 mm)	Weft Density (yarn per 100 mm)	Reed Counts (reed dent per 100 mm)	Yarn Counts Per Reed	Total Number of Warp Yarns
zigzag	10, 17, 24	60	1181	30	6	360

**Table 2:** Material properties of the zigzag-shaped 3D woven composites obtained from the simulation.

Heights/mm	E_11_/ GPa	E_22_/ GPa	E_33_/ GPa	V_12_	V_23_	V_13_	G_12_/ GPa	G_13_/ GPa	G_23_/ GPa
10	32	9.79	9.79	0.21	0.21	0.3	9.56	9.56	21
17	149.9	44.5	44.5	0.22	0.22	0.3	43.45	43.45	95.6
24	158.9	47.1	47.1	0.25	0.25	0.3	46.06	46.06	101.34
